# Causal effects of walking pace on osteoarthritis: a two-sample mendelian randomization study

**DOI:** 10.3389/fgene.2023.1266158

**Published:** 2023-10-11

**Authors:** Peng Qiu, Junyu Wu, Lihong Kui, Mingxian Chen, Shuaibing Lv, Zhongkai Zhang

**Affiliations:** ^1^ Department of Rehabilitation, First Affiliated Hospital of Wenzhou Medical University, Wenzhou, Zhejiang, China; ^2^ School of Physical Education, Shanghai University of Sport, Shanghai, China; ^3^ Xiamen Rehabilitation Hospital, Xiamen, China; ^4^ School of Exercise and Health, Shanghai University of Sport, Shanghai, China

**Keywords:** walking pace, osteoarthritis, causal relationship, mendelian randomization, genome-wide association studies

## Abstract

**Background:** Osteoarthritis (OA) is one of the most common joint diseases worldwide, imposing a substantial burden on individuals and society. Numerous pieces of evidence suggest that walking pace (WP) can serve as a predictive indicator for the risk of various diseases, and observational studies have also found a potential link between WP and the risk of OA. However, the causal relationship between WP and the risk of OA remains unclear.

**Methods:** We conducted a mendelian randomization (MR) study using data from the European Genome-wide Association Study, which included WP (including 459,915 participants), OA (including 10,083 cases and 40,425 controls), knee OA (including 24,955 cases and 378,169 controls), and hip OA (including 15,704 cases and 378,169 controls). Single nucleotide polymorphisms (SNPs) associated with WP were utilized to infer causal associations with OA and its subtypes. The Inverse Variance Weighted (IVW) technique served as the primary causal analysis method. Three auxiliary MR methods - MR-Egger, weighted median, and maximum likelihood - were used to substantiate the IVW results. Sensitivity analyses were performed to examine heterogeneity and pleiotropy. In addition, multivariate MR (MVMR) analysis was used to assess causality after adjustment for three potential confounders.

**Results:** According to the results of the IVW method, every 1 standard deviation increased in genetic WP corresponds to an 89% reduction in the risk of OA (odds ratio (OR) = 0.11; 95% confidence interval (CI), 0. 06–0.19; *p* = 1.57 × 10^−13^), an 83% reduction in the risk of knee OA (OR = 0.17; 95% CI, 0.11–0.28; *p* = 2.78 × 10^−13^), and a 76% reduction in the risk of hip OA (OR = 0.24; 95% CI, 0.14–0.43; *p* = 1.51 × 10^−6^). These results were confirmed by the three additional MR methods and validated by the sensitivity analysis. Ultimately, the MVMR analysis confirmed that the role of WP in reducing the risk of OA and its subtypes remains consistent regardless of potential confounders.

**Conclusion:** The results of our MR study highlight a significant causal association between WP and the susceptibility to OA, including its knee and hip subtypes. These findings propose that WP could be utilized as a potential prognostic factor for OA risk.

## 1 Introduction

Osteoarthritis (OA) is increasingly acknowledged as a prevalent degenerative joint disease, bearing significant implications for both individuals and society (Hunter et al., 2014; Martel-Pelletier et al., 2016; Yue and Berman, 2022). OA is characterized by the deterioration of one or multiple joints, including of both small (e.g., hand) and large joints (e.g., hip and knee). The burden posed by OA is expressed through a range of symptoms, including pain, morning stiffness, and joint crepitus. These symptoms can compromise joint stability and potentially result in disability (Martel-Pelletier et al., 2016; Mandl, 2019; Kolasinski et al., 2020). The global prevalence of OA has been reported to exceed 300 million people (GBD, 2017 Disease and Injury Incidence and Prevalence Collaborators, 2018), making it a major contributor to disability in the aging population. The socioeconomic impact of OA is substantial, with annual healthcare costs surpassing 330 billion dollars due to the high prevalence and associated expenses (Murphy et al., 2018). Given the typically gradual progression of OA, early and accurate prediction or identification of OA risk plays a pivotal role in its prevention and management, alleviating the burden on individuals and society (Chu et al., 2012; Mahmoudian et al., 2021).

Walking pace (WP) is commonly regarded as the sixth vital sign, alongside heart rate, body temperature, respiration, pain, and blood pressure (Fritz and Lusardi, 2009). It serves as a valuable parameter for assessing an individual’s physical function and overall health status (Fonseca Alves et al., 2017). In addition, WP has emerged as a significant predictor of various health outcomes and disease risks. Recent studies have demonstrated that a decline in WP is associated with an increased risk of cardiovascular disease (Fonseca Alves et al., 2017; Veronese et al., 2018), disability (Perera et al., 2016), cognitive impairments (Quan et al., 2017), and type-2 diabetes (Boonpor et al., 2022). While the prognostic value of WP has been acknowledged (Bejek et al., 2006; Tanaka et al., 2017), its specific impact on the risk of developing OA within the sports medicine context remains to be fully elucidated.

This study utilized a two-sample mendelian randomization (MR) approach to comprehensively investigate the possible association between WP and the risk of OA, including its subtypes: knee OA and hip OA (Burgess et al., 2019). MR analysis harnesses independent single nucleotide polymorphisms (SNPs) as instrumental variables (IVs) due to their robust correlation with the exposures, facilitating the estimation of causal relationships between exposures and outcomes (Burgess et al., 2017). This method efficiently mitigates the influence of confounders, eliminates reverse causation, and minimizes bias. By implementing MR analysis, this study aims to ascertain the potential role of WP as a simplistic predictive indicator for OA risk and its subtypes. This research has the potential to provide cost-effective strategies for predicting OA risk in clinical practice and establishing the foundation for targeted preventive interventions.

## 2 Materials and methods

### 2.1 Study design

**FIGURE 1:**
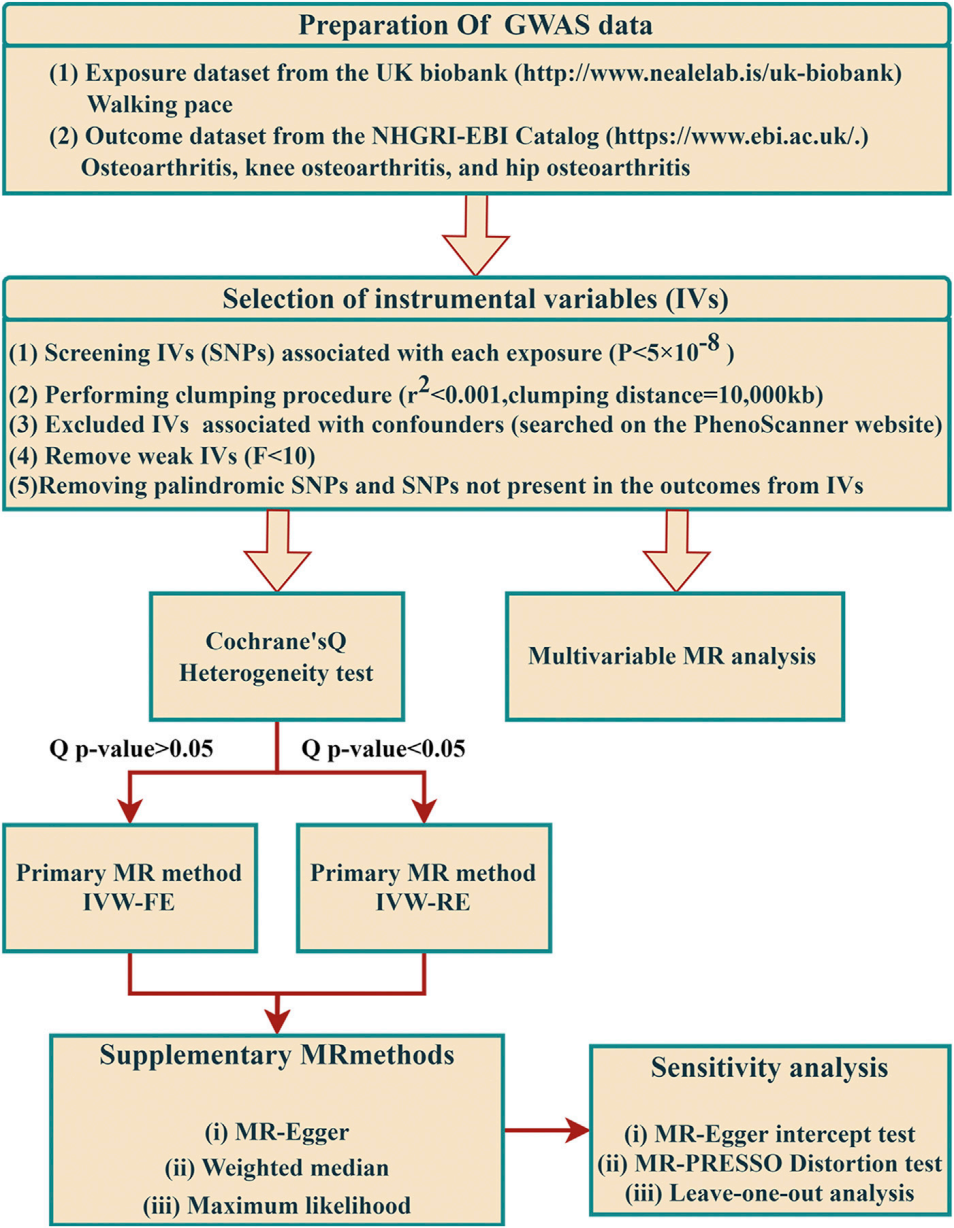
The flow framework of MR analysis shows the causal effect of walking pace on osteoarthritis. SNP, single-nucleotide polymorphism.

To evaluate the causal effect of WP on OA, a two-sample MR design was used (Figure 1). This MR study is predicated on three fundamental assumptions: First, the selected genetic IVs must have a strong association with WP. Second, the chosen IVs should be unaffected by confounders that could potentially influence the relationship between WP and OA. Lastly, the IVs are assumed to exert influence on OA risk solely through WP (Emdin et al., 2017). The data employed in our research is accessible for download. All the European Genome-wide Association Study (GWAS) incorporated in this study received ethical approval from their respective institutions.

### 2.2 Data sources

The summary data for WP in the GWAS were derived from the publicly available United Kingdom Biobank database, encompassing 459,915 European participants, designated under the GWAS-ID “ukb-b-4711” (Bycroft et al., 2018). WP was evaluated based on the participant’s responses to the query, How would you categorize your regular walking speed? (A pace of less than 3 miles per hour is considered slow, 3-4 miles per hour corresponds to a moderate, steady pace, whereas a speed exceeding 4 miles per hour would be classified as fast). This question was part of a touchscreen questionnaire (https://biobank.ndph.ox.ac.uk/showcase/field.cgi?id=924). All participants, excluding those who reported an inability to walk, provided responses. Detailed information can be found in Table 1.

**TABLE 1:** Comprehensive details on the GWAS datasets employed in this MR study.

Dataset type	Item	GWAS ID	Author	Consortium	Year	Population	Sample size	PMID
Exposure	Walking pace	ukb-b-4711	Ben Elsworth	MRC-IEU	2018	European	459,915	NA
	Smoking	ieu-b-4877	Liu M	GSCAN	2019	European	607,291	30643251
	Alcohol consumption	ukb-b-5779	Ben Elsworth	MRC-IEU	2018	European	462,346	NA
	Diabetes	ukb-b-10753	Ben Elsworth	MRC-IEU	2018	European	461,578	NA
Outcome	Osteoarthritis	ebi-a-GCST005814	Zengini E	NA	2018	European	10,083 cases and 40,425 controls	29559693
	Knee osteoarthritis	ebi-a-GCST007090	Tachmazidou I	NA	2019	European	24,955 cases and 378,169 controls	30664745
	Hip osteoarthritis	ebi-a-GCST007091	Tachmazidou I	NA	2019	European	15,704 cases and 378,169 controls	30664745

GWAS, genome-wide association study; MRC-IEU, Medical Research Center-Integrative Epidemiology Unit; GSCAN, GWAS & Sequencing Consortium of Alcohol and Nicotine; MR, mendelian randomization; NA, not applicable.

Genome-wide meta-analysis data (Zengini et al., 2018; Tachmazidou et al., 2019) provided the GWAS statistics for OA (10,083 cases and 40,425 controls), knee OA (24,955 cases and 378,169 controls), and hip OA (15,704 cases and 378,169 controls). The diagnosis of OA was premised on both self-reported information and Hospital Episode Statistics data, with emphasis on disease specificity to certain joints (Zengini et al., 2018). Self-reported OA was defined by affirmative responses to the question, “Has a doctor ever informed you of any serious medical conditions or disabilities?” on a self-administered touch-screen questionnaire. Following this, disease coding data was collected through a computer-aided personal interview. The United Kingdom Biobank uses the International Classification of Diseases-10 (ICD-10) code to classify hospital-diagnosed OA, extracted from Hospital Episode Statistics data. Identification of knee and hip OA cases hinged on the clinical necessity for joint replacement or radiographic evidence of disease (Kellgren-Lawrence grade ≥2) (Tachmazidou et al., 2019). Detailed information can be referred to in Table 1.

Analyses were adjusted for smoking, alcohol consumption, and diabetes by applying multivariate MR (MVMR). The IEU Open GWAS project provided data for these exposures. Detailed information is presented in Table 1.

### 2.3 Selection of instrumental variables

This MR study used SNPs that exhibited robust associations with WP as IVs. We employed a genome-wide significance level (*p* < 5 × 10^−8^) for the IV selection process in the MR analysis (Vaucher et al., 2018). To ascertain the individual independence of each IV, we imposed constraints on the linkage disequilibrium (LD) correlation coefficient at *r* ^2^ < 0.001 and defined the clumping window size to be greater than 10,000 kb. Additionally, we conducted a search using PhenoScanner (http://www.phenoscanner.medschl.cam.ac.uk/) to pinpoint SNPs linked with potential confounding factors like smoking and alcohol consumption (with a significance threshold of *p* < 5 × 10^−8^). Any such SNPs were subsequently excluded from our selected instrumental variables (see Supplementary Table S1 for more details).

During the harmonization stage of aligning exposure and outcome statistics, we discarded palindromic and incompatible SNPs from the IVs, in addition to those SNPs absent in the outcome GWAS summary data. Following this, we calculated the F-statistic of the IVs employing the formula F = Beta^2^ exposure/SE^2^ exposure (Lawlor et al., 2008) to evaluate the robustness of the IVs and curtail the effect of weak instrument bias on the causality interpretation. We only retained IVs exhibiting an F-statistic exceeding 10 to minimize the bias introduced by weak IVs (Pierce et al., 2011).

### 2.4 Statistical analyses

To scrutinize the potential causal link between WP and OA, our MR study principally employed the IVW method within its analytical construct (Burgess et al., 2013). The selection between a fixed-effect IVW (IVW-FE) or a random-effect IVW (IVW-RE) was contingent upon the results from Cochrane’s Q heterogeneity test. In instances of detected heterogeneity (*p* < 0.05), we employed the IVW-RE model, which delivers a more conservative estimate. Conversely, when no heterogeneity was identified, the fixed-effect IVW model was implemented (Greco et al., 2015). The IVW method, based on meta-analysis principles, has been extensively validated for making causal inferences in MR studies (Pagoni et al., 2019).

To strengthen the credibility and establish the directionality of our results, we utilized three additional MR methods [MR-Egger, weighted median, and maximum likelihood] for causal association assessments. The MR-Egger regression presumes that more than 50% of IVs undergo horizontal pleiotropy (Bowden et al., 2015). On the other hand, the weighted median method presumes that horizontal pleiotropy is present in fewer than 50% of IVs (Bowden et al., 2016). The maximum likelihood approach, which optimizes the likelihood function to estimate the parameters of a given probability distribution, typically results in smaller standard errors. (Milligan, 2003). We acknowledged only those exposure-outcome pairs with uniform directional implications across all MR methods as possessing a causal association.

To verify the robustness of our Mendelian randomization findings, we implemented an array of sensitivity analyses. We initially employed the MR-Egger intercept to identify potential horizontal pleiotropy (Bowden et al., 2015; Verbanck et al., 2018). Moreover, we employed the MR-PRESSO distortion test, an integral component of the MR-PRESSO framework, to ascertain whether MR estimates remained consistent post the elimination of potential pleiotropic outliers (Verbanck et al., 2018). Subsequently, we performed leave-one-out sensitivity analyzes to determine whether MR estimates were unduly affected by any single SNP.

A *p*-value less than 0.05 was set as the threshold for statistical significance. Causal association results were presented as odds ratios (OR) accompanied by their respective 95% confidence intervals (95% CI). The analyses were performed using the “TwoSampleMR” (version 0.5.6) and “MRPRESSO” (version 1.0) packages within the R programming platform (version 4.2.3).

### 2.5 Multivariable mendelian randomization analysis

Besides univariable MR, a MVMR approach was undertaken. The potential confounders including smoking, alcohol consumption, and diabetes. After merging the GWAS datasets of exposure and confounders, we ensured that each IV retained a strong correlation (*p* < 5 × 10^−8^) with at least one exposure or confounder. We pruned SNPs within a 10,000 kb window using a threshold of *r* ^2^ < 0.001 to mitigate LD. Causal effects were assessed using the IVW approach after adjusting for these confounders and removing palindromes and SNPs not found in the resulting GWAS data.

## 3 Results

### 3.1 Selection of instrumental variables

**FIGURE 2:**
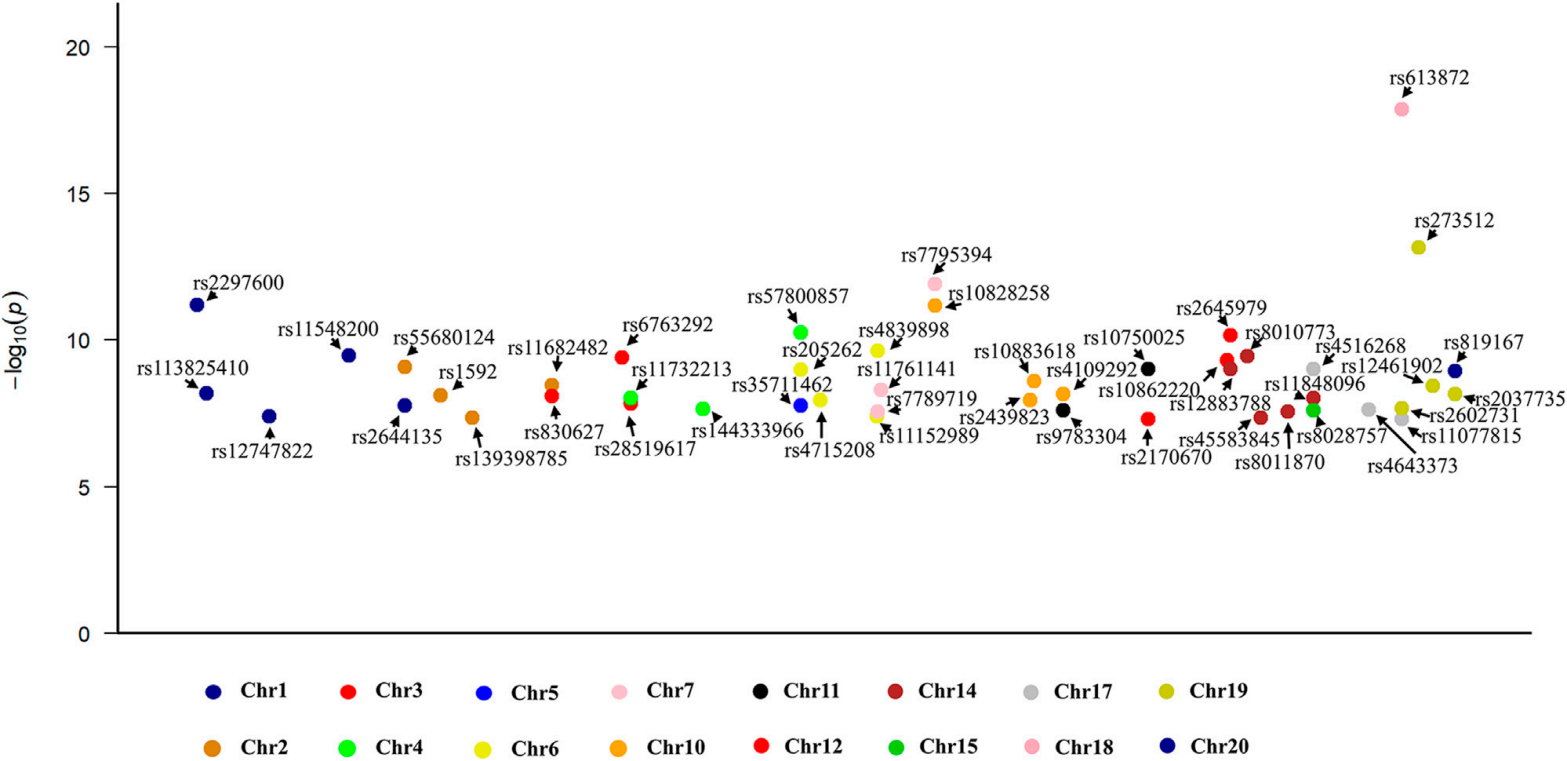
A Manhattan plot illustrating the 47 single nucleotide polymorphisms identified as instrumental variables within the exposure dataset.

In the WP assessment, a total of 47 independent SNPs were selected as IVs (detailed in Supplementary Table S2). Figure 2 shows a Manhattan plot of these SNPs. These instrumental SNPs explained 0.14% of the variance of WP. The F statistic of each individual SNPs ranges from 29.79 to 77.51, indicating adequate instrument strength.

### 3.2 Causal relationship between WP and OA

**FIGURE 3:**
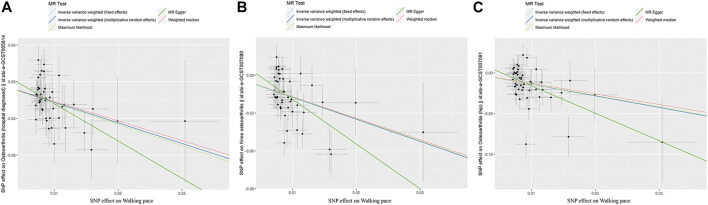
Scatter plot of genetic associations between exposure and outcome. **(A)** walking pace and osteoarthritis. **(B)** walking pace and knee osteoarthritis. **(C)** walking pace and hip osteoarthritis.

Through Cochrane’s Q test, there was heterogeneity in WP with knee OA and hip OA, then the random-effect IVW method was used (*p* < 0.05, Table 2). The MR result of IVW showed that for every 1 standard deviation genetically increased walk pace has an 89% lower risk of OA (OR = 0.11; 95% CI, 0.06–0.19; *p* = 1.57 × 10^−13^), an 83% lower risk of knee OA (OR = 0.17; 95% CI, 0.11–0.28; *p* = 2.78 × 10^−13^), a 76% lower risk of hip OA (OR = 0.24; 95% CI, 0.14–0.43; *p* = 1.51 × 10^−6^) (Table 3; Figure 3), in the meantime, the results of other MR methodologies were consistent with those obtained using the IVW approach.

**TABLE 2:** Results of sensitivity analyses.

Exposure	Outcome	Cochran’s Q test				Pleiotropy			
		MR-egger		IVW		MR egger		MR-PRESSO	
		Q	Q-pval	Q	Q-pval	Intercept	*p*-value	n Outliers	*p*-value
WP	OA	44.337	0.457	46.216	0.422	0.017	0.179	NA	NA
WP	Knee OA	79.189	0.001	84.677	0.000	0.016	0.084	NA	NA
WP	Hip OA	77.180	0.002	81.270	0.001	0.018	0.130	1	0.557

WP, walking pace; OA, osteoarthritis; IVW, inverse variance weighted; SE, standard error.

**TABLE 3:** Mendelian randomization assessment of walking pace’s causal influence on osteoarthritis.

Exposure	Outcome	n SNP	Method	OR (95% CI)	*p*-value
WP	OA	46	IVW-RE	0.11 (0.06.0.19)	3.26E-13
			IVW-FE	0.11 (0.06.0.19)	1.57E-13
			MR Egger	0.02 (0.00.0.25)	4.77E-03
			Weighted median	0.12 (0.05.0.28)	1.99E-06
			Maximum likelihood	0.10 (0.05.0.18)	1.28E-13
WP	Knee OA	47	IVW-RE	0.17 (0.11.0.28)	2.78E-13
			IVW-FE	0.17 (0.12.0.25)	3.74E-23
			MR Egger	0.03 (0.00.0.22)	1.45E-03
			Weighted median	0.18 (0.11.0.30)	1.26E-10
			Maximum likelihood	0.18 (0.12.0.25)	1.43E-20
WP	Hip OA	47	IVW-RE	0.24 (0.14.0.43)	1.51E-06
			IVW-FE	0.24 (0.16.0.37)	1.62E-10
			MR Egger	0.03 (0.00.0.44)	1.26E-02
			Weighted median	0.27 (0.14.0.53)	1.03E-04
			Maximum likelihood	0.25 (0.16.0.39)	1.41E-09

WP, walking pace; OA, osteoarthritis; IVW-RE, inverse variance weighted (random effects); IVW-FE, inverse variance weighted (fixed effects); OR, odds ratios; CI, confidence intervals.

### 3.3 Sensitivity analysis

In the sensitivity assessment, excluding Cochrane’s Q test, the findings of the MR-Egger intercept test indicated an absence of significant horizontal pleiotropy influencing the MR analysis (*p* > 0.05; Table 2). In addition, MR-PRESSO identified 1 outlier (rs11732213) in the hip osteoarthritis analysis, and the association remained even after this SNP was removed. Finally, the leave-one-out sensitivity evaluation corroborated the robustness of the outcomes, with none of the SNPs significantly altering the results when eliminated (Supplementary Figure S1).

### 3.4 Results of multivariable mendelian randomization analysis

**FIGURE 4:**
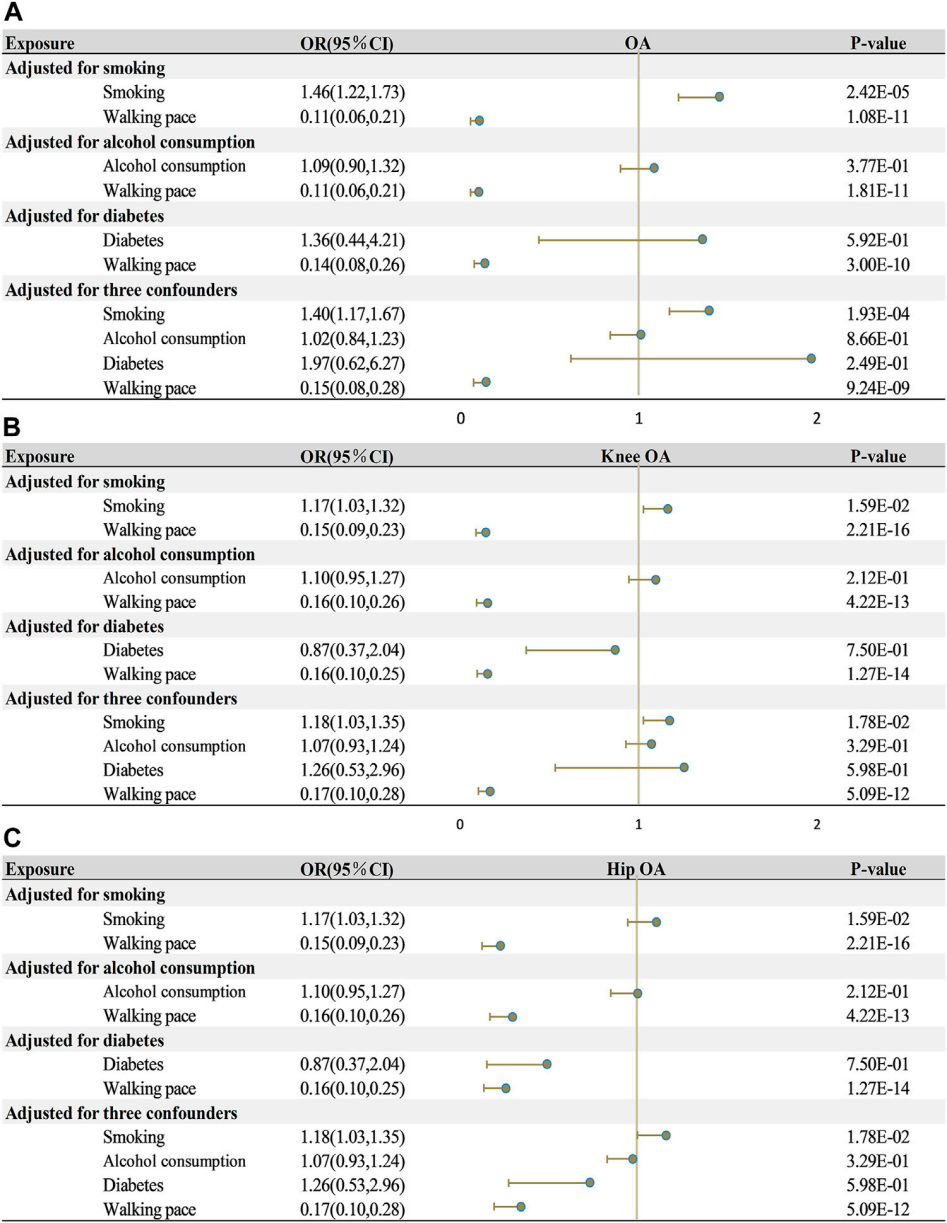
Multivariate mendelian randomization analysis of the effects of exposure on outcome. **(A)** walking pace on osteoarthritis. **(B)** walking pace on knee osteoarthritis. **(C)** walking pace on hip osteoarthritis. OA, osteoarthritis; OR, odds ratios; CI, confidence intervals.

In order to elaborate on the associations between WP and OA and its two subtypes, we executed MVMR analysis, with an adjustment for three confounding variables (smoking, alcohol consumption, and diabetes; Figure 4). No individual factor could modify the causal effect of WP and OA and its two subtypes (all *p* < 0.05). Even when all factors were simultaneously adjusted for, the significant causal connection between walking pace and osteoarthritis, including its two subtypes, was sustained (*p* < 0.001).

## 4 Discussion

Our findings from the MR study highlight substantial causal links between WP and the risks associated with overall OA, as well as its subtypes - knee and hip OA. In detail, the IVW mendelian randomization outcomes demonstrated that a genetically driven increase in WP by 1 standard deviation is linked with a significant reduction (89%) in the risk of developing OA (OR = 0.11; 95%CI, 0.06–0.19). This evidence underscores the potential of WP to serve as a predictive factor for OA risk.

The association observed in our study, demonstrating an inverse relationship between WP and the risks of OA, knee OA, and hip OA, agrees with prior research. Numerous prospective cohort and observational investigations, which included long-term follow-up of middle-aged and elderly populations, scrutinized the link between WP and the risk of knee and hip OA. These studies consistently report that a reduction in WP is significantly associated with an increased risk of OA (Purser et al., 2012), knee OA (Purser et al., 2012; Fenton et al., 2018; Master et al., 2020; Harkey et al., 2021), and hip OA (Tanaka et al., 2017). Remarkably, this association was observed in both symptomatic knee and hip OA, as well as in radiographic OA diagnoses (Purser et al., 2012). Much of the existing research has focused on the association between WP and knee OA risk, providing evidence that a reduction in WP heightens the risk of knee replacement surgery in the ensuing year (Harkey et al., 2021). Moreover, studies have identified an association between decreased WP and elevated mortality due to knee OA, wherein every decrease of 0.2 m/s in WP corresponds to a 23% surge in mortality risk (Master et al., 2020). Yet, the observational and prospective cohort study designs inherently limit the ability to infer causality between WP, knee OA, and hip OA. Any definitive claim about the causal relationship among these factors would be presumptive. To navigate these limitations, our study harnessed the two-sample MR method, proficient at minimizing biases intrinsic to study designs. SNPs associated with WP were selected as IVs for MR, which was critical for our analysis (Emdin et al., 2017). Using SNPs allows us to understand individual genetic susceptibility to WP and its impact on OA while minimizing confounding bias typical of observational studies. And strengthens our causal inferences, highlighting the robustness of our findings and the potential of WP as a predictor of OA risk. This methodology allowed us to gain deeper insights into the causal links between WP, knee OA, and hip OA, thereby fortifying the validity of our findings.

While some studies have investigated the relationship between WP and the risk of OA, the mechanisms underlying the association between a decline in WP and increased OA risk remain uncertain (Fenton et al., 2018; Harkey et al., 2021). This complexity arises from the multifactorial nature of WP as an indicator of overall physical function and the presence of multiple risk factors associated with OA (Mobasheri et al., 2017; Hunter and Bierma-Zeinstra, 2019). Consequently, elucidating the potential mechanisms connecting WP and OA poses a challenge. Several potential mechanics may contribute to the association between decreased WP and increased OA risk. Firstly, from a biomechanical perspective, the faster WP or slower WP influences the mechanical parameters of the entire lower limb. Abundant research evidence indicates that faster WP leads to reduced knee extension during the swing phase of walking (Arnold et al., 2007). Concurrently, a faster WP contributes to a shorter stance phase in the gait cycle (60.2% for fast WP compared to 62.6% for slow WP), potentially diminishing the load on the joints (Landry et al., 2007). Additionally, studies reveal that older adults have relatively heightened muscle and tendon activity, and co-activation in the knee and ankle joints during fast walking (Robbins and Maly, 2009). This could increase joint load or possibly provide more joint stability, hence offering better buffering (Arnold et al., 2007). Secondly, WP to a certain extent reflects an individual’s lower limb muscle strength. A faster WP signifies greater joint torque and strength (Chiu and Wang, 2007), which holds potential benefits for OA prevention (Øiestad et al., 2022). Thirdly, WP is closely linked to an individual’s physical activity level. Evidence shows that physical activity of certain intensities plays a positive role in preventing or mitigating OA symptoms (Kraus et al., 2019). Moderate physical activity promotes joint health by enhancing lubrication, and muscle strength (Wallis et al., 2013). In contrast, factors such as obesity and inflammation associated with increased sedentary time and decreased physical activity levels have been confirmed as significant risk factors for OA (Martel-Pelletier et al., 2016; Yue and Berman, 2022). Finally, recent findings suggest that inflammation is a crucial element in the pathogenesis of OA (Martel-Pelletier et al., 2016). It's noteworthy that WP shows good sensitivity to other chronic diseases (Middleton et al., 2015). Some of these diseases could have co-morbidities or interdependencies due to shared pathological mechanisms. For example, inflammation might underlie the pathogenesis of several chronic diseases, including OA. Consequently, from a predictive perspective, a deceleration in WP could serve as an early indication of OA. When joint wear and degradation begin, yet without the manifestation of apparent symptoms, WP may already be impacted.

Our study carries several critical strengths. Primarily, we are the first to utilize MR to explore the causal relationship between WP and OA, which includes knee and hip OA. Crucially, we analyzed results for OA, and its subtypes, to gain a comprehensive understanding of the complex relationship between WP and OA risk in different joints. Significantly, after accounting for potential confounders like smoking, alcohol consumption, and diabetes, the causal link between WP and OA and its subtypes continues to hold. Additionally, our MR analysis draws from substantial GWAS datasets, which lends credibility to our findings due to the large participant count involved. Moreover, stringent criteria are implemented for instrument variable selection, ensuring that only causal relationships confirmed by multiple MR methods are deemed credible. To bolster the robustness of the findings, an array of two sample MR methods is employed, accompanied by a comprehensive set of sensitivity analyses. Lastly, the potential of WP as a preliminary screening tool for OA, knee OA, and hip OA is identified, presenting a promising avenue for cost reduction in healthcare. Moreover, WP measurement provides individuals with valuable insights into their functional status and overall health, paving the way for targeted exercise interventions and health promotion initiatives. Consequently, this study represents a significant stride at the intersection of sports, exercise science, and epidemiology, advancing our understanding of WP and its implications for health.

Despite the strengths of our study, several limitations should be acknowledged. Firstly, after meticulous examination of the GWAS summary data from European populations, the study results are most directly applicable to this group. Caution must be exercised when extrapolating these findings to other racial and ethnic groups, such as Asian and African populations. This is due to potential significant differences in genetic structure, lifestyle factors, and environmental exposures among different populations, which could influence the observed associations. We will continue to monitor updates and developments in this field, seeking available GWAS databases from diverse populations to provide a more comprehensive analysis and insight. Secondly, due to the inherent constraints of GWAS summary data, stratified analyses based on common demographic variables such as gender and age were not feasible. This limitation is not unique to our study. We searched many databases and conducted a review of current MR studies (Bountziouka et al., 2022; Chen et al., 2022; Dempsey et al., 2022) related to WP, revealing common methodological limitations. And did not present exhaustive patient characteristics. This suggests a broader challenge in the field, possibly due to database constraints or the nature of GWAS data collection. It appears to be a common challenge for researchers in the field. Additionally, it is important to note that WP is influenced by multiple factors, including other chronic conditions, nutrition, physical activity, and more, which may be associated with the risk of OA or unrelated to it. The definitive determination of OA still necessitates medical examinations and imaging techniques.

## 5 Conclusion

Our MR study presents substantial evidence underlining a significant causal association between WP and the risk of OA, including knee and hip OA. Specifically, a reduction in WP corresponds with increased susceptibility to OA, knee OA, and hip OA. These findings potentially advocate for the utilization of WP as a predictive determinant for OA, knee OA, and hip OA risk. This can aid in the early identification of OA risk through a straightforward measure, thereby mitigating the individual and societal impact of OA progression.

## Data Availability

The original contributions presented in the study are included in the article/Supplementary Material, further inquiries can be directed to the corresponding author.
