# Supplementary material for: Causal effects of walking pace on osteoarthritis: a two-sample mendelian randomization study
Source: Front Genet. 2023 Oct 11;14:1266158. doi: 10.3389/fgene.2023.1266158 (PMC10598773; doi:10.3389/fgene.2023.1266158)
Supplement: Supplementary file 2 [file Image1.pdf]

Supplementary Figures

1 Supplementary Figures

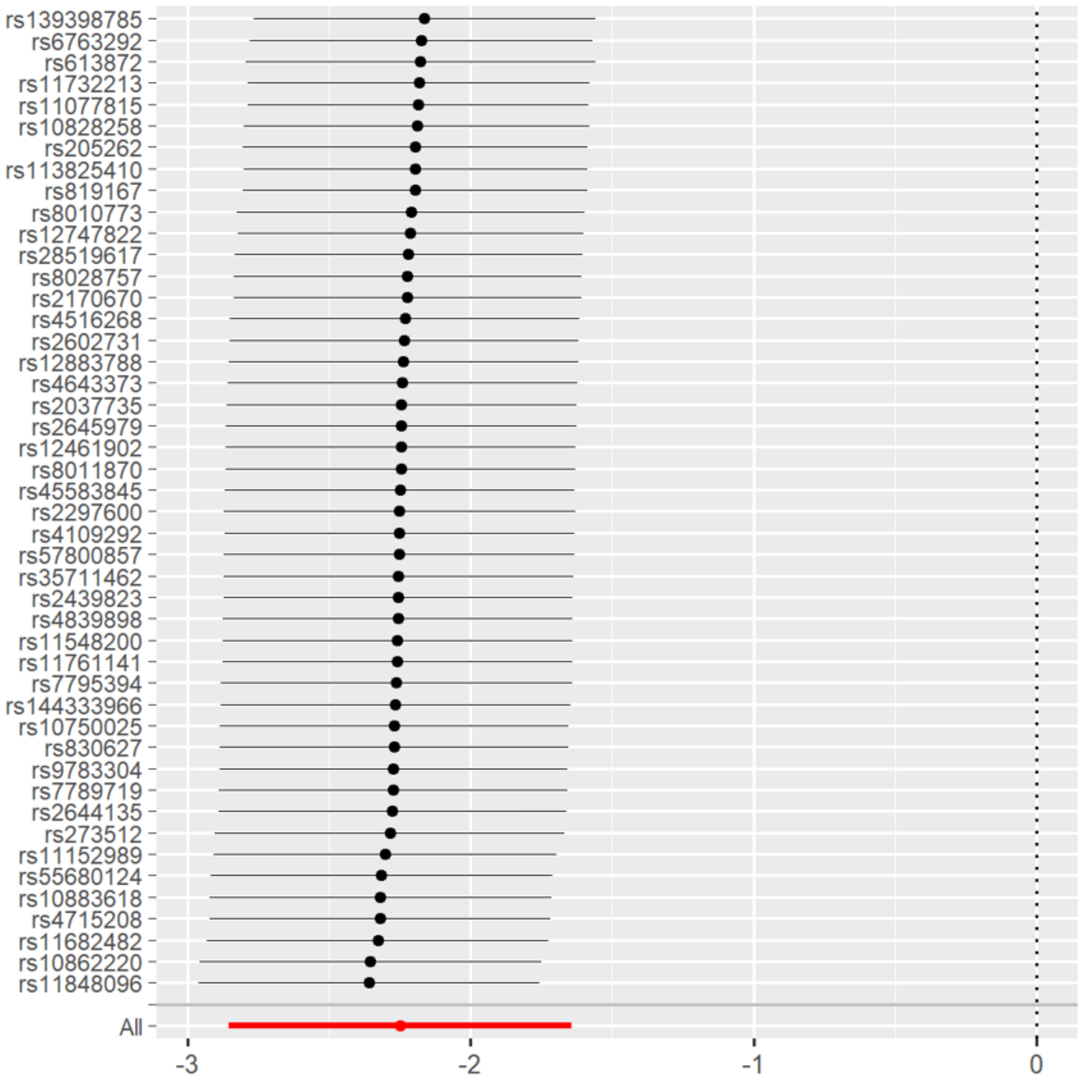

MR leave-one-out sensitivity analysis for walking pace on osteoarthritis

(A)

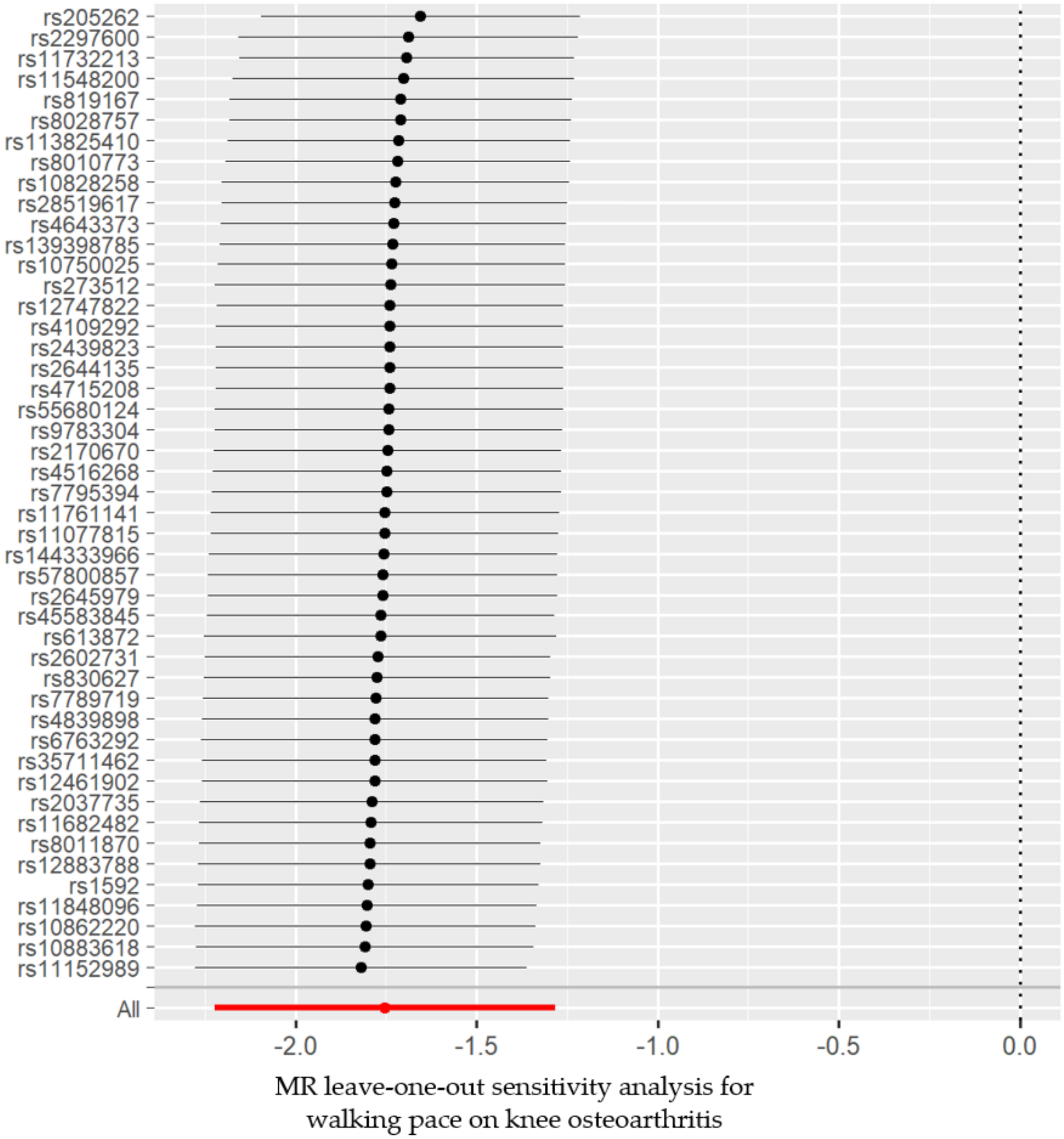
rs205262
rs2297600
rs11732213
rs11548200
rs819167
rs8028757
rs113825410
rs8010773
rs10828258
rs28519617
rs4643373
rs139398785
rs10750025
rs273512
rs12747822
rs4109292
rs2439823
rs2644135
rs4715208
rs55680124
rs9783304
rs2170670
rs4516268
rs7795394
rs11761141
rs11077815
rs144333966
rs57800857
rs2645979
rs45583845
rs613872
rs2602731
rs830627
rs7789719
rs4839898
rs6763292
rs35711462
rs12461902
rs2037735
rs11682482
rs8011870
rs12883788
rs1592
rs11848096
rs10862220
rs10883618
rs11152989
All
-2.0
-1.5
-1.0
-0.5
0.0
MR leave-one-out sensitivity analysis for
walking pace on knee osteoarthritis

(B)

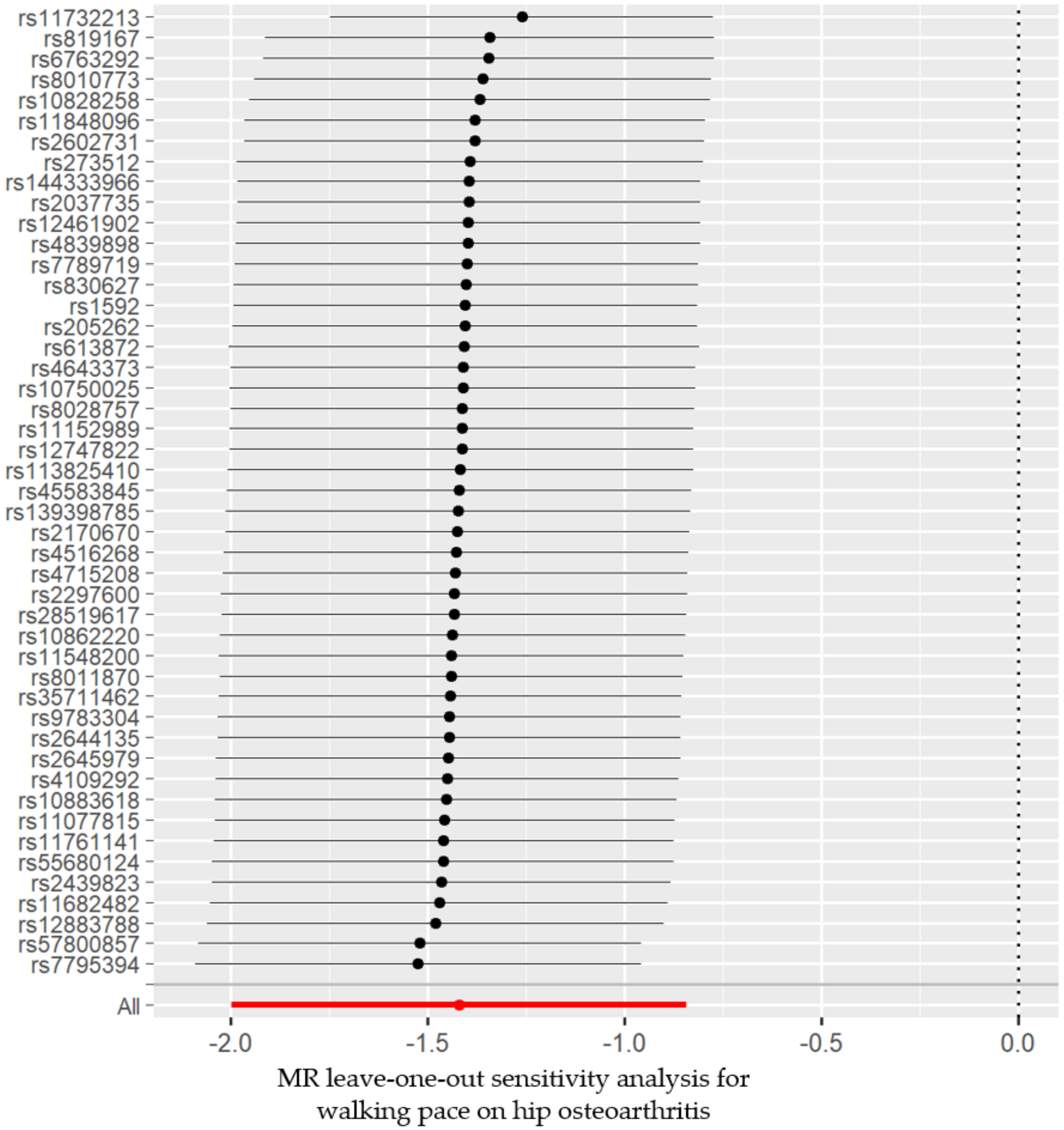

(C)

Supplementary Figure S1. Leave-one-out sensitivity analysis under genome-wide significance threshold instrumental variables: (A) walking pace on osteoarthritis; (B) walking pace on knee osteoarthritis; (C) walking pace on hip osteoarthritis.
